# The Resilience of Attitude Toward Vaccination: Web-Based Randomized Controlled Trial on the Processing of Misinformation

**DOI:** 10.2196/52871

**Published:** 2024-12-04

**Authors:** Benoît Béchard, Julie A Gramaccia, Dominique Gagnon, Elhadji Anassour Laouan-Sidi, Ève Dubé, Mathieu Ouimet, Delphine de Hemptinne, Sébastien Tremblay

**Affiliations:** 1 School of Psychology Université Laval Québec, QC Canada; 2 Department of Communication University of Ottawa Ottawa, ON Canada; 3 Quebec National Institute of Public Health Québec, QC Canada; 4 Department of Anthropology Université Laval Québec, QC Canada; 5 Department of Political Science Université Laval Québec, QC Canada

**Keywords:** attitude toward vaccination, misinformation, reinformation, confidence, perceived tentativeness, vaccine hesitancy, COVID-19

## Abstract

**Background:**

Before the COVID-19 pandemic, it was already recognized that internet-based misinformation and disinformation could influence individuals to refuse or delay vaccination for themselves, their families, or their children. Reinformation, which refers to hyperpartisan and ideologically biased content, can propagate polarizing messages on vaccines, thereby contributing to vaccine hesitancy even if it is not outright disinformation.

**Objective:**

This study aimed to evaluate the impact of reinformation on vaccine hesitancy. Specifically, the goal was to investigate how misinformation presented in the style and layout of a news article could influence the perceived tentativeness (credibility) of COVID-19 vaccine information and confidence in COVID-19 vaccination.

**Methods:**

We conducted a web-based randomized controlled trial by recruiting English-speaking Canadians aged 18 years and older from across Canada through the Qualtrics (Silver Lake) paid opt-in panel system. Participants were randomly assigned to 1 of 4 distinct versions of a news article on COVID-19 vaccines, each featuring variations in writing style and presentation layout. After reading the news article, participants self-assessed the tentativeness of the information provided, their confidence in COVID-19 vaccines, and their attitude toward vaccination in general.

**Results:**

The survey included 537 participants, with 12 excluded for not meeting the task completion time. The final sample comprised 525 participants distributed about equally across the 4 news article versions. Chi-square analyses revealed a statistically significant association between general attitude toward vaccination and the perceived tentativeness of the information about COVID-19 vaccines included in the news article (*χ*^2^_1_=37.8, *P*<.001). The effect size was small to moderate, with Cramer *V*=0.27. An interaction was found between vaccine attitude and writing style (*χ*^2^_1_=6.2, *P*=.01), with a small effect size, Cramer *V*=0.11. In addition, a Pearson correlation revealed a significant moderate to strong correlation between perceived tentativeness and confidence in COVID-19 vaccination, *r*(523)=0.48, *P*<.001. The coefficient of determination (*r*^2^) was 0.23, indicating that 23% of the variance in perceived tentativeness was explained by confidence in COVID-19 vaccines. In comparing participants exposed to a journalistic-style news article with those exposed to an ideologically biased article, Cohen *d* was calculated to be 0.38, indicating a small to medium effect size for the difference in the perceived tentativeness between these groups.

**Conclusions:**

Exposure to a news article conveying misinformation may not be sufficient to change an individual’s level of vaccine hesitancy. The study reveals that the predominant factor in shaping individuals’ perceptions of COVID-19 vaccines is their attitude toward vaccination in general. This attitude also moderates the influence of writing style on perceived tentativeness; the stronger one’s opposition to vaccines, the less pronounced the impact of writing style on perceived tentativeness.

**International Registered Report Identifier (IRRID):**

RR2-10.2196/41012

## Introduction

### Background

The COVID-19 pandemic has heightened misinformation about vaccines [1,2]. According to the World Health Organization, the world was not only facing a pandemic but also an infodemic [3], where an excessive amount of information—including true, false, and misleading information—is readily available and may hinder the ability of individuals to determine appropriate actions regarding preventive measures, including vaccination [4,5]. The propagation of misinformation concerning COVID-19 vaccination has raised concerns about vaccine safety and effectiveness, arguments against the necessity of vaccination, and considerations of morality and freedom [6]. Among others, Puri et al [7] argued that disseminating misinformation may contribute to lower-than-expected vaccine uptake rates [8-11].

Exposure to misinformation, whether through social media, traditional media, or other sources, can lead to the formation of false beliefs, distortions in memory, and influence decision-making processes [12]. Left uncorrected, misinformation can persist and continue to affect individuals’ reasoning even after being presented with corrective information [13]. The strength of misinformation is such that sometimes attempts to correct it through retractions can have the opposite effect and, paradoxically, reinforce the misbelief [14]. In the context of public policy, misinformation can have harmful effects by influencing individual beliefs, attitudes, and behaviors and ultimately impacting public policy decisions [15]. The impact of misinformation is not limited to individual decision-making but can extend to societal decisions that may counter the community’s best interests [14]. Misinformation can also contribute to public distrust in science and influence choices that lead to potentially harmful outcomes such as vaccine hesitancy, inappropriate use of medical treatments, or neglect of preventive health measures, posing a threat to public safety [16].

### Previous Work

Despite the lack of consensus in the scientific literature, some researchers suggest that sociodemographic attributes, such as age, sex, income level, education, and political orientation, may influence susceptibility to misinformation about COVID-19 vaccines [6-8]. Younger adults who are undecided about getting vaccinated for COVID-19 may be more likely to do so if exposed to prosocial and altruistic messages [12]. Low-income demographics and individuals not adhering to COVID-19 governmental guidelines may be especially vulnerable to web-based misinformation, decreasing vaccine acceptance [13]. In contrast, higher numeracy skills and trust in scientists would be associated with lower susceptibility to COVID-19–related misinformation [1]. In addition to trust placed in the scientific community, confidence in vaccination itself would be a significant factor in the acceptance of COVID-19 vaccines [17,18]; confidence is intricately intertwined with epistemic uncertainty, as communicating uncertainties stemming from incomplete (and still evolving) knowledge can impact the level of trust in factual information about both the pandemic and COVID-19 vaccines [19].

### Reinformation and Misinformation

The developing nature of information regarding COVID-19 vaccines creates an opening for misinformation through reinformation media. “Reinformation” presents itself as an alternative to traditional media [20]. Reinformation, as defined in the context of the hybrid media system where traditional media coexist and interact with digital media, refers to the proactive, politically motivated, and ideologically driven aspect of information. Unlike misinformation, which characterizes the nature of information, reinformation is embodied in producing, using, and sharing content. It involves the community-driven creation and dissemination of information that aligns with specific political agendas and ideologies [21]. Reinformation encompasses actively generating and disseminating information influenced by partisan motivations, ideological perspectives, practical experiences, and evolving knowledge frameworks. It underscores the dynamic nature of information production and dissemination in various domains, highlighting the continuous interplay between existing knowledge, emerging insights, and societal influences. Reinformation sources usually act as brokers that deliver counter-official narrative news intending to influence readers into adopting specific viewpoints [22]. Contents disseminated under reinformation are presented to mimic traditional news reporting [23], emulating the writing style, structure, and presentation commonly found in conventional news articles.

Although reinformation, by definition, entails polarizing information produced by self-appointed news organizations [20,21], this process frequently extends beyond the media realm. For example, in health sciences, updates and new perspectives on diseases, treatments, and health care practices can reinform the current framework of medical knowledge. By incorporating alternative viewpoints, this framework evolves and adjusts to mirror the shifting landscape of health care [24], potentially yielding positive or negative outcomes on the public perception and understanding of an issue. Ngai et al [25] stress that imitating mainstream news or scientific reports contributes to the proliferation of disinformation (ie, the state in which a person may find themselves when exposed to misinformation spread by alternative reinformation media). Based on a content analysis of 140 Facebook (Meta) posts, their study reveals that adopting the language features of traditional sources can result in higher engagement on social media platforms (ie, likes, comments, and shares). Reinformation, while not inherently false, can sometimes exhibit biases that promote the adoption of a specific viewpoint [20], potentially contributing to the fueling of COVID-19 vaccine hesitancy [26].

### Communication Strategies

Communication-based strategies [27,28], fact-checking [29-31], prebunking [32], and debunking [33] have been proposed as promising approaches to counter the detrimental effects of misinformation, including reinformation, during “infodemics.” These techniques—ranging from disclaimers and misinformation warnings, awareness-raising and educational videos, the use of “scare tactics” (eg, disease images), to algorithmic approaches for identifying trends of “fake news” in content—aim to provide a means of evaluating the accuracy and truthfulness of claims [34], and to convey the weight-of-evidence regarding vaccines and related myths [28]. Notably, providing accurate information and correcting misinformation have been found to help reduce the ongoing impact of fake news and counter health misinformation [35-37]. However, the effectiveness of fact-checking platforms and debunking strategies may be limited when confronted with polarizing, emotionally charged information [38], given that the primary emphasis of fact-checking lies in identifying false content rather than addressing biased or misleading information [32]. While not necessarily untrue, ideologically biased information can also sway people’s perceptions and decision-making about COVID-19 vaccination, depending on how it is summarized and reported [33]. For instance, if information is presented in a manner that creates uncertainty about the evidence, it can impact the credibility of the information being communicated [39]. Conversely, presenting consensual evidence may positively influence the attitude toward the subject matter addressed by the findings [40]. Thus far, research on reinformation and the role of ideologically biased information in vaccine hesitancy have been somewhat overshadowed by the focus on false claims [32,41].

The prevalence of polarizing content shared by reinformation sources poses a challenge to preventing and controlling pandemics such as COVID-19. In an infodemic, where information quality is inconsistent and rapidly changing across media platforms, the writing style of discourse and the layout of the information may contain varying levels of emotional charge and ideological biases. Depending on how messages are shared with the public, information can adversely impact readers’ attitudes toward COVID-19 vaccination [42]. Addressing the impact of reinformation requires understanding how information influenced by ideology affects vaccine hesitancy. Public health communication strategies need to do more than just provide facts; they must also address people’s deeply held beliefs and biases that influence their views. It is crucial to consider how existing attitudes toward vaccination can shape individuals’ understanding and response to vaccine-related information. These efforts can help combat misinformation and promote informed decision-making about vaccines.

### Aim of This Study

The present study explored how readers perceive information regarding COVID-19 vaccines, considering the influence of the writing style and the format through which the information is presented. Specifically, we investigated whether there could be an association between the framing of information within a news article published on the web and individuals’ perceptions of the tentativeness surrounding COVID-19 vaccine information and their level of confidence in opting for vaccination against COVID-19.

### Research Hypotheses

Based on the existing literature, we formulated the following research hypotheses:

First, hypothesis 1 (H1_1_) that participants’ perceived tentativeness of information about COVID-19 vaccines will vary significantly based on the writing style of the news articles, with ideologically biased articles leading to a higher level of perceived tentativeness than articles adhering to journalistic standards.

Second, hypothesis 2 (H2_1_) that participants’ confidence in COVID-19 vaccination will positively correlate with their attitudes toward vaccines. Participants with a more positive attitude toward vaccines will show higher confidence levels in COVID-19 vaccination.

## Methods

### A Web-Based Experimental Survey

We conducted a web-based randomized controlled experimental survey. We intentionally manipulated the writing style and presentation layout to assess their effect on the perceived tentativeness of information shared about COVID-19 vaccines and confidence in COVID-19 vaccination. We considered the general attitude toward vaccination as a potential confounding factor influencing both variables under investigation (ie, perceived tentativeness and confidence).

### Data Collection

The experiment was conducted using the Qualtrics (Silver Lake) paid opt-in panel system between November 7 and November 23, 2022. The system randomly selects participants from various market research panels and web-based platforms. Qualtrics panel system uses stratified sampling randomization to ensure that each subgroup is comparable in terms of sex, age, highest level of education achieved, and geographic location. Respondents join a Qualtrics panel by filling out a form and agreeing to participate in surveys. Incentives come in the form of points, which can be redeemed for vouchers, gifts, or other types of compensation. The topic of the study is not disclosed in the invitation to prevent biases. The sample is nonprobabilistic and based on voluntary participation, although Qualtrics randomly selects from a pool of potential respondents.

The experiment aimed to differentiate the association of ideologically biased material from that of journalistic style-based material about (1) the perceived tentativeness of information shared about COVID-19 vaccines, (2) confidence in COVID-19 vaccines, and (3) the general attitude toward vaccination (whether against COVID-19 or other viruses). To ensure representativeness, the panel was benchmarked against known census targets, including age, region, sex, and education. Individuals aged 18 years and older, who self-identified as English-speaking Canadians, resided in 1 of the 10 Canadian provinces at the time of the study, and had access to a computer connected to the internet, were eligible to participate in the experiment. Prospective respondents from the northern territories (ie, Yukon, Northwest Territories, and Nunavut) were not included due to concerns about internet connectivity, access reliability, and speed. Participants were drawn from the English-speaking Canadian population to minimize the possibility of translation bias (ie, the alteration of findings that can occur when the research material or the findings themselves are translated from one language to another). Participants who spent less than 247 seconds (4 min 6 s) completing the survey were excluded from the sample, as this was considered insufficient time for a comprehensive understanding of the purpose of the study. A question was added to the questionnaire to identify and exclude participants with color blindness. This was necessary because the experimental design used distinct colored infographics (ie, vaccine administration by qualified personnel vs zooming in on a needle) to differentiate between journalistic and nonjournalistic layouts.

### Outcome Measures

The primary outcomes, which were self-assessed using questionnaires, consisted of 2 measures, that is, the perceived tentativeness of information shared about COVID-19 vaccines and confidence in COVID-19 vaccination. Perceived tentativeness refers to the subjective impression or belief that the information conveyed is uncertain, provisional, or not fully confirmed. This concept was measured through a 7-item questionnaire (eg, “The information in the document is definitive” and “Based on this document, our understanding of COVID-19 vaccine side effects is complete”) drawn from Kimmerle et al [33], which used a 5-point Likert scale (ranging from strongly disagree to strongly agree), where higher scores indicated less tentativeness.

Confidence in COVID-19 vaccination was assessed by querying respondents’ opinions on current vaccines using an 8-item questionnaire based on Kimmerle et al [33]. The questionnaire used a 5-point Likert-type scale, ranging from strongly disagree to strongly agree. The items included (1) “COVID-19 vaccine is promising,” (2) “COVID-19 vaccine is safe,” (3) “COVID-19 vaccine is certainly helpful,” (4) “The risks related to COVID-19 vaccine are lower than the benefits,” (5) “If a loved one had a need for which COVID-19 vaccine is one of the solutions, I would like him or her to benefit from it,” (6) “If I had a need for which COVID-19 vaccine is one of the solutions, I would like to benefit from it,” (7) “COVID-19 vaccine roll-out is not concerning,” and (8) “Currently available vaccines are the most effective way to combat the COVID-19 epidemic.” In this case, higher scores indicated greater confidence.

The general attitude toward vaccination was measured on a 10-point rating “slider” scale ranging from highly provaccine to highly antivaccine using the question, “Here is a 10-point scale on which the views that people might hold are arranged from extremely pro-vaccine (1) to extremely anti-vaccine (10). Where would you place yourself on this scale?” The rating of the general attitude scale was then reversed to facilitate the interpretation of findings. Participants were not informed of the concepts being measured to ensure the concealment of the outcomes and avoid subject bias (ie, the potential influence of participants on the results due to their previous knowledge of the concepts measured). Each outcome was measured after the participant read the news article. All questionnaires used in the study are available in Multimedia Appendix 1.

### Study Design

**Figure 1:**
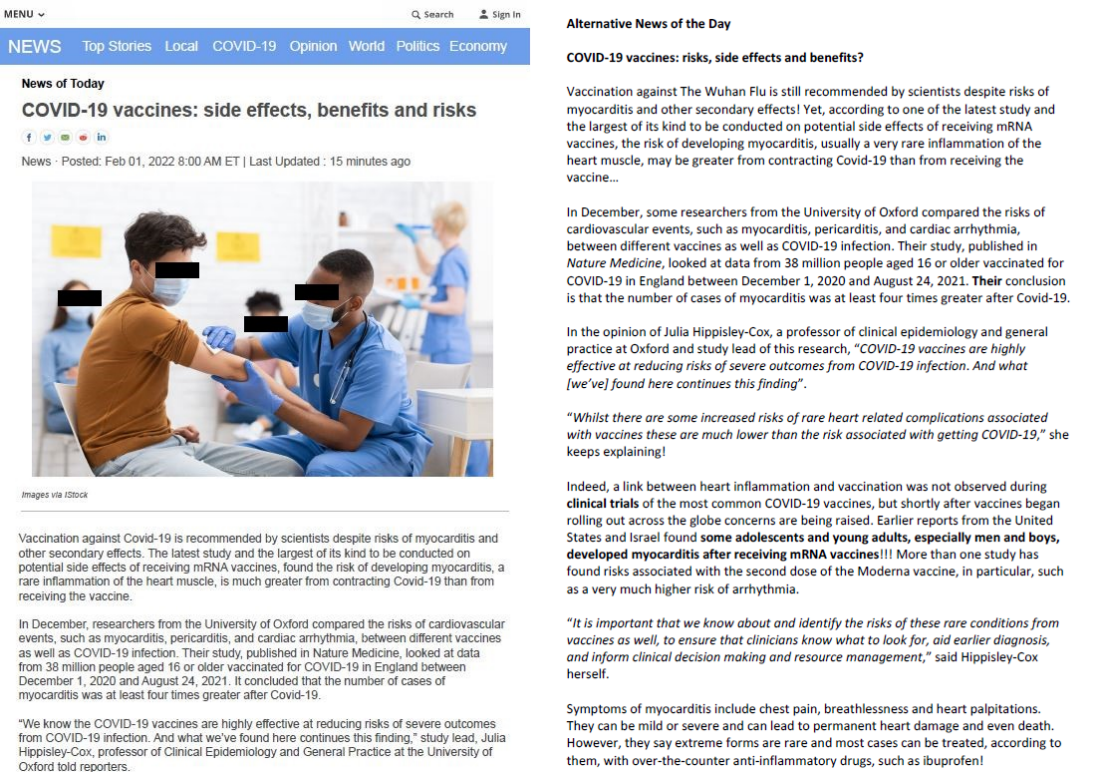
Examples of the news article on COVID-19 mRNA vaccine side effects, benefits, and risks, written in a journalistic style with corresponding layout (left; version 1), and of the news article written in an ideologically-biased style without adherence to journalistic layout principles (right; version 4).

In collaboration with an experienced journalist from a prominent English-language news outlet in Canada, holding a bachelor’s degree (bachelor of arts) in journalism from one of the leading English-speaking universities in Canada, and with over a decade of journalism experience, we cocreated a news article reporting on the potential side effects, benefits, and risks of COVID-19 mRNA vaccination (Figure 1).

A total of 4 versions of the news article were developed by manipulating the writing style (journalistic vs ideologically biased) and the presentation layout (journalistic layout vs no journalistic layout; refer to Multimedia Appendices 2-5 for all versions). In the third and fourth versions, the original text was edited using linguistic and discursive choices such as grammatical structure, vocabulary, and rhythm of words. In the journalistic writing style, the text emphasized the recommendation for COVID-19 vaccination, acknowledging potential risks like myocarditis and other side effects. The text referenced the latest and most extensive study of mRNA vaccine side effects to establish its credibility. The ideologically biased text was crafted to portray COVID-19 vaccines as posing substantial risks, including myocarditis and other side effects. The mention of the infrequent yet existing risks of heart-related complications following vaccination could inadvertently instill concerns among readers regarding vaccine safety (refer to Multimedia Appendices 2-5 to review the 4 artifacts as they were presented to participants). These alterations allowed the text to align with a biased style of discourse.

The experimental design was a 2×2 factorial (between-group) design. This design involved 2 factors—writing style and presentation layout. Each factor had 2 variations; the “writing style” factor had levels of journalistic and ideologically biased while the “presentation layout” factor had levels of journalistic layout with colored graphs and no journalistic layout (plain). All possible combinations of these variations were tested (full factorial design). This approach allowed us to examine the main effects of each factor and the interaction effects between writing style and presentation layout on the outcome variables. The original design was strictly adhered to and followed consistently throughout the study. Participants were randomly assigned to read 1 of the following 4 versions of the news articles:

1. Group JWJL (journalistic writing style and journalistic layout) with colored graphs,

2. Group JWNL (journalistic writing style and no journalistic layout; plain),

3. Group IdWJL (ideologically biased writing style and journalistic layout) with colored graphs, and

4. Group IdWNL (ideologically biased writing style and no journalistic layout; plain).

### Procedure

Participants agreed to participate in the experiment after completing the consent form. Each participant was required to provide sociodemographic information, including age, province of residence, the highest level of education attained, and primary language, before receiving the intervention. Qualtrics’ block randomizer for equal group size assigned participants equally across all 4 conditions while ensuring allocation concealment. Participants were not informed of their group assignment. They were instructed to read and review the assigned news article carefully, paying close attention to details and information. Each participant read only 1 version of the news article, and their blindness to experimental manipulation was maintained by ensuring that the version presented to them was unpublished. Emphasis was placed on reading the document in its entirety before accessing the survey questions, as revisiting it after proceeding was not permitted. Participants completed the questionnaires on perceived tentativeness and confidence immediately after reading the news article. Subsequently, participants were required to disclose their stance on vaccination in general, not just mRNA COVID-19 vaccines.

The survey was completed by 537 participants, with 12 excluded from the sample due to their task completion time not meeting the inclusion criterion. All data that could have been regarded as outliers were retained in the final sample, considering the inherently “positional” nature of the topic under investigation at the time of the study (in this regard, refer to the work of Nicholson [43]). Imputation techniques to address attrition were planned but not used, as they were considered unnecessary due to the small number of observations removed from the analysis sample. To avoid biased responses resulting from a carry-over effect, we intentionally withheld the true nature of the study at the start of the questionnaire. Therefore, participants who wished to withdraw their consent had the opportunity to do so by selecting the option “I do not consent to participate in this research,” which was presented at the end of the study.

### Data Analysis

An item-level reliability analysis was carried out on the perceived tentativeness and confidence questionnaires. Both measures elicited good to excellent internal consistency, with Cronbach α values of 0.84 and 0.94 (≥0.70), respectively. These coefficients suggested that both questionnaires had a high level of reliability for assessing the perceived tentativeness of information shared on COVID-19 vaccines and confidence in COVID-19 vaccination. A composite numerical score was then calculated for both outcome variables. The structure of the questionnaires enabled the use of a scoring system that relies on adding up the included items (or calculating the average) [33,40]. We performed descriptive analyses of the sample, randomization checks, and conducted chi-square analyses (some with interaction terms) to examine how the writing style and presentation layout influence both outcome variables, with consideration of the participant’s general attitude toward vaccination. The strength of the association between the perceived tentativeness and the general attitude toward vaccination was assessed using Cramer *V* for the chi-square analyses. We also calculated Cohen *d* to assess the effect size of the difference in perceived tentativeness between groups. All analyses were conducted on an intent-to-treat basis, and the statistical significance was defined as *P*<.05. Data were analyzed using SAS (version 9.4; SAS Institute).

### Ethical Considerations

The study was approved by Université Laval’s Research Ethics Committee (CERUL: 2022-007). The study was part of a protocol for a multicomponent research initiative registered on October 17, 2022, under the International Registered Report Identifier (IRRID): DERR1-10.2196/41012 (due to its classification outside the scope of a clinical trial, the study was not registered separately as such). The trial was reported in accordance with the CONSORT (Consolidated Standards of Reporting Trials) checklist [44] (refer to Multimedia Appendix 6 for the CONSORT checklist). Informed consent was obtained from all participants, and they were provided with the option to opt out at any time during the study (refer to Multimedia Appendix 7 for the consent form). To protect the confidentiality of participants, we excluded their names from any reports, used randomly assigned numbers to anonymize research data, and limited access to research materials to only the principal investigator on a secure server at Université Laval. Individual results were kept private and not disclosed. The study was supported by a grant from the Canadian Institutes of Health Research (award GA3177725).

## Results

### Characteristics of the Sample

**Figure 2:**
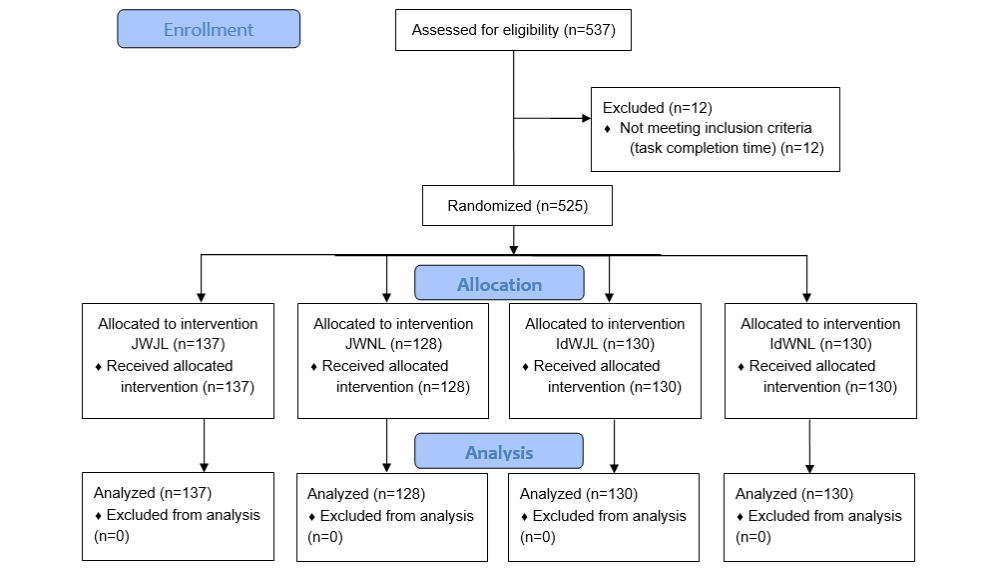
CONSORT (Consolidated Standards of Reporting Trials) flow diagram. A total of 12 participants were excluded from the study after their task completion times did not meet the inclusion criteria among 537 assessed for eligibility. IdWJL: ideologically-biased writing style and journalistic layout with colored graphs; IdWNL: ideologically-biased writing style and no journalistic layout; JWJL: journalistic writing style and journalistic layout with colored graphs; JWNL: journalistic writing style and no journalistic layout.

The final sample included 525 participants exposed to 1 of the 4 conditions. Participants took between 4 minutes 36 seconds and 16 minutes 28 seconds (median 8 min 43 s, SD 4 min 14 s) to complete the survey. The distribution of participants’ characteristics is shown in Table 1. Through random assignment, the characteristics of the participants closely mirrored those of the English-speaking Canadian population in terms of age, geographical location, sex, and highest level of education achieved. Among the 525 participants, 290 were female, making up 55.2% of the sample. The largest age group was individuals aged 65-74 years, with 148 participants out of 525 (28.2%). Furthermore, 117 (22.3%) participants had finished their high-school education, 107 (20.4%) had completed college studies, 91 (17.3%) held an associate degree, 156 (29.7%) had commenced undergraduate studies, and 54 (10.3%) had achieved a master’s or PhD degree. The 4 experimental groups were equated according to the sociodemographic characteristics (as outlined in Table 1), and the distribution of participants among the groups was almost equal in numbers, with only a slight variation due to the time criterion. Specifically, the 2 groups receiving ideologically biased summaries (IdWJL and IdWNL) had 130 participants each (24.8%), while the 2 groups receiving journalistic summaries (JWJL and JWNL) had 137 participants (26%) and 128 participants (24.4%), respectively (refer to Figure 2 for the CONSORT flow diagram).

Table 2 presents the descriptive statistics for the perceived tentativeness of information shared about COVID-19 vaccines, confidence in COVID-19 vaccination, and general attitude toward vaccination across the 4 experimental groups. Participants exposed to the news article with an ideologically biased style reported lower mean perceived tentativeness scores, regardless of whether the article had a journalistic layout with colored graphs (mean 21.7, SD 5.4) or without a journalistic layout (mean 22.1, SD 4.9). This suggests that participants in groups IdWJL and IdWNL perceived the information as slightly more tentative and provisional, irrespective of the layout. The average scores for confidence in COVID-19 vaccination were similar across all 4 groups, showing only marginal differences. This suggests that trust in vaccination was consistent across the groups, with no significant variations observed despite differences in writing style and layout. Regarding the general attitude toward vaccination, participants across all groups generally displayed high scores, reflecting a generally positive attitude toward vaccination, with means ranging from 7.4 (SD 2.9) to 7.8 (SD 2.3).

**Table 1:** Participants’ baseline characteristics and outcome measures across groups (N=525).

Variable			Group JWJL^a^, n (%)		Group JWNL^b^, n (%)		Group IdWJL^c^, n (%)		Group IdWNL^d^, n (%)		Overall, %		Perceived tentativeness, mean (SD)		Confidence, mean (SD)
**Sex**															
	Male	53 (38.7)		57 (44.5)		61 (46.9)		63 (48.5)		44.8		22.9 (5.1)		31.1 (7.4)	
	Female	83 (60.6)		71 (55.5)		69 (53.1)		67 (51.5)		55.2		22.7 (4.6)		30.6 (6.6)	
	Prefer not to disclose	1 (0.7)		—^e^		—		—		—		—		—	
**Age group (year)**															
	18-34	13 (9.5)		16 (12.5)		16 (12.3)		9 (6.9)		10.3		22.9 (5)		30.3 (6.3)	
	35-44	28 (20.4)		22 (17.2)		12 (9.2)		15 (11.5)		14.7		21.8 (4.6)		28.8 (7.2)	
	45-54	20 (14.6)		23 (17.9)		18 (13.9)		12 (9.2)		13.9		22.2 (5.5)		30.4 (6.5)	
	55-64	34 (24.8)		29 (22.7)		29 (22.3)		40 (30.8)		25.1		22.7 (4.8)		31 (7)	
	65-74	32 (23.4)		29 (22.7)		44 (33.8)		43 (33.1)		28.2		23 (4.8)		31.3 (7.2)	
	More than 75	10 (7.3)		9 (7)		11 (8.5)		11 (8.5)		7.8		24.8 (3.6)		33.5 (6.2)	
**Education**															
	High school	22 (16.1)		28 (21.9)		29 (22.3)		38 (29.2)		22.3		22.6 (4.7)		30.9 (4.7)	
	College	33 (24.1)		23 (18)		27 (20.8)		24 (18.5)		20.4		23.6 (4.7)		31.1 (4.7)	
	Associate degree	24 (17.5)		26 (20.3)		21 (16.1)		20 (15.4)		17.3		22.5 (5.4)		30.3 (8.3)	
	Undergraduate	45 (32.8)		42 (32.8)		36 (27.7)		33 (25.4)		29.7		22.5 (4.9)		31.1 (6.7)	
	Graduate	13 (9.5)		9 (7)		17 (13.1)		15 (11.5)		10.3		22.7 (4.5)		31.4 (6.2)	
**Geographical location**															
	Atlantic^f^	17 (12.4)		14 (10.9)		11 (8.5)		11 (8.4)		10.1		22.9 (5.4)		31.8 (5.9)	
	British Columbia	28 (20.4)		24 (18.8)		25 (19.2)		25 (19.2)		19.4		22.9 (4.5)		30.7 (6.4)	
	Ontario	61 (44.5)		50 (39.1)		62 (47.7)		59 (45.4)		44.2		22.7 (5)		31.1 (7.2)	
	Quebec	6 (4.4)		9 (7)		7 (5.4)		8 (6.2)		5.7		23.4 (4)		30.2 (7.7)	
	Western^g^	25 (18.3)		31 (24.2)		25 (19.2)		27 (20.8)		20.6		22.4 (4.9)		30.2 (7.1)	

^a^JWJL: journalistic writing style and journalistic layout with colored graphs.

^b^JWNL: journalistic writing style and no journalistic layout.

^c^IdWJL: ideologically biased writing style and journalistic layout with colored graphs.

^d^IdWNL: ideologically biased writing style and no journalistic layout.

^e^Not applicable.

^f^Atlantic: New Brunswick, Newfoundland and Labrador, Nova Scotia, and Prince Edward Island.

^g^Western (Prairies): Alberta, Saskatchewan, and Manitoba.

**Table 2:** Perceived tentativeness, confidence, and general attitude toward vaccination across groups (N=525).

Variable	n (%)	Perceived tentativeness, mean (SD)	Confidence, mean (SD)	General attitude, mean (SD)
Group JWJL^a^	137 (26.1)	23.6 (4.6)	31.3 (6.1)	7.8 (2.3)
Group JWNL^b^	128 (24.4)	23.7 (4.2)	31.4 (6.1)	7.9 (2.4)
Group IdWJL^c^	130 (24.8)	21.7 (5.4)	30 (7.2)	7.7 (2.4)
Group IdWNL^d^	130 (24.8)	22.1 (4.9)	30.5 (8.2)	7.4 (2.9)

^a^JWJL: journalistic writing style and journalistic layout with colored graphs.

^b^JWNL: journalistic writing style and no journalistic layout.

^c^IdWJL: ideologically biased writing style and journalistic layout with colored graphs.

^d^IdWNL: ideologically biased writing style and no journalistic layout.

### The Perceived Tentativeness of Information Shared About COVID-19 Vaccines

**Figure 3:**
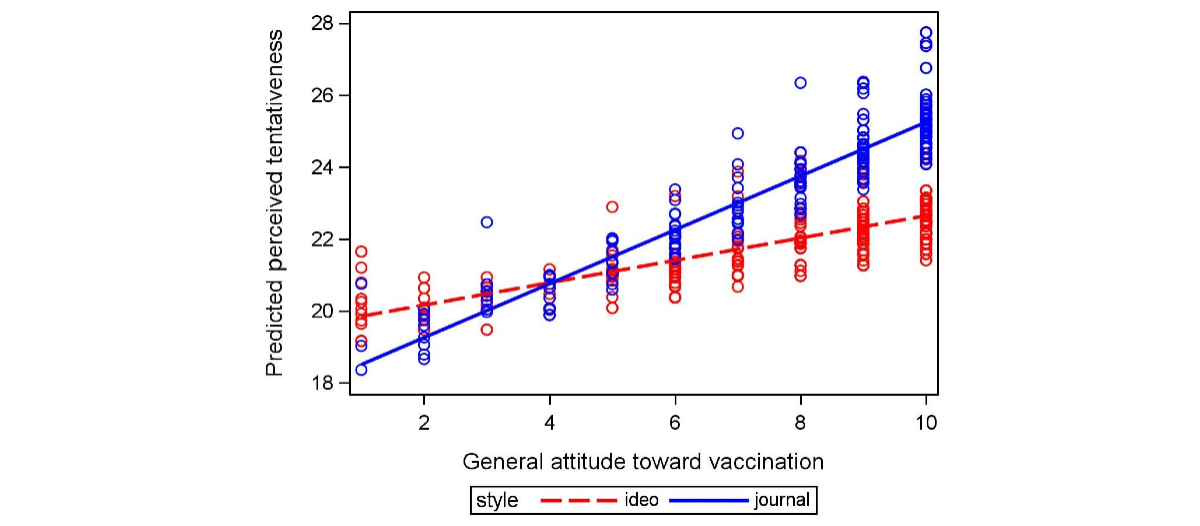
Interaction between the general attitude toward vaccination and writing style on perceived tentativeness.

We first conducted chi-square analyses to examine the relationship between writing style, presentation layout, and participants’ perceived tentativeness of information shared about COVID-19 vaccines’ side effects. The results indicated that the perceived tentativeness significantly differed based on the general attitude toward vaccination (*χ*^2^_1_=37.8, *P*<.001), suggesting that participants who held a more favorable view toward vaccines perceived information as less tentative. The effect size was small to moderate (Cramer *V*=0.27), indicating a modest association between vaccine attitude and perceived tentativeness. In addition, there was a significant interaction between the general attitude toward vaccination and writing style in influencing perceived tentativeness (*χ*^2^_1_=6.2, *P*=.01; Figure 3). Participants who held a more favorable attitude toward vaccination, in general, perceived the information as less tentative when it was presented in a journalistic style. The effect size was small (Cramer *V*=0.11). There was no significant association between presentation layout and perceived tentativeness (*P*=.48), and no significant association or interaction was observed for participants’ age, sex, and level of education. The calculation of Cohen *d* for group JWJL compared with group IdWJL revealed an effect size of 0.38, indicating a small to medium effect. This suggests that the perceived tentativeness of the information was moderately influenced by the writing style, with a notable difference between the group exposed to the journalistic writing style and the one exposed to the ideologically biased writing style.

### Confidence in COVID-19 Vaccination

**Figure 4:**
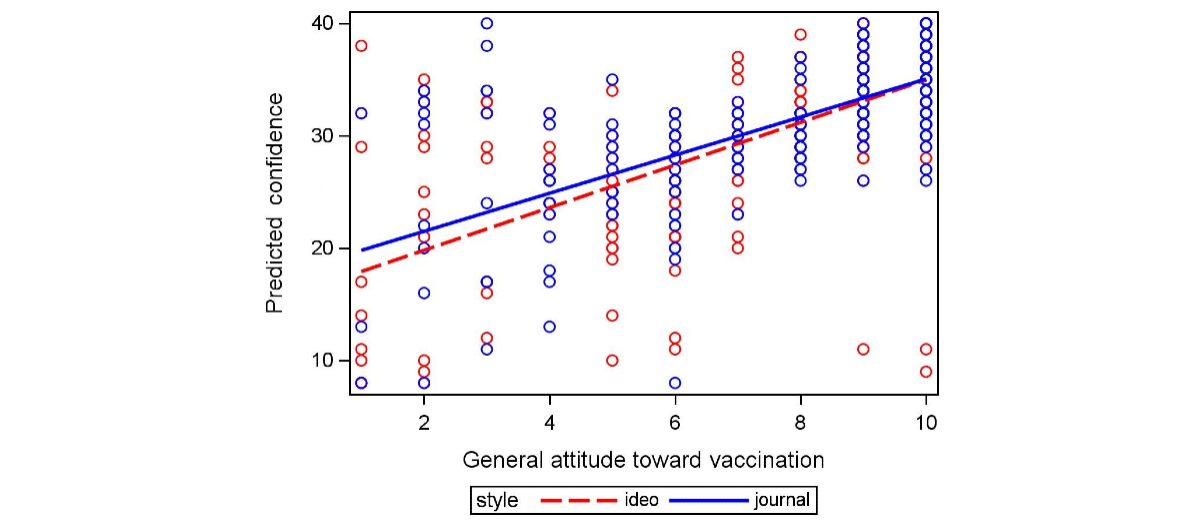
Interaction between the general attitude toward vaccination and writing style on confidence.

We then conducted chi-square analyses to assess participants’ confidence in COVID-19 vaccination. Confidence significantly differed based on the general attitude toward vaccination (*χ*^2^_1_=296.8, *P*<.001; Figure 4), suggesting that participants with a more favorable view of vaccines, in general, tended to express greater confidence in COVID-19 vaccination. There was no statistically significant difference in confidence based on either writing style or presentation layout. Similarly, demographic characteristics showed no significant association with confidence in COVID-19 vaccines.

### Perceived Tentativeness and Confidence in COVID-19 Vaccines

An exploratory Pearson correlation coefficient was calculated to examine the association between perceived tentativeness and confidence in COVID-19 vaccines. The results revealed a significant moderate-to-strong correlation [*r*(523)=0.48, *P*<.001], indicating that there is a positive linear relationship wherein higher levels of perceived tentativeness regarding information about COVID-19 vaccines correspond to increased confidence in COVID-19 vaccination. The coefficient of determination (*r*^2^) was used to quantify the proportion of variance in perceived tentativeness explained by the confidence in COVID-19 vaccination. The effect size was *r*^2^=0.23, indicating a moderate level of association between the 2 variables.

## Discussion

### Principal Findings

Our findings revealed that a prevailing factor associated with the perception of COVID-19 vaccines is people’s attitude toward vaccination in general. The general attitude toward vaccination moderated the relationship between the writing style and perceived tentativeness. Specifically, individuals with negative attitudes toward vaccines tend to perceive COVID-19 vaccine information in a journalistic style as more tentative. Conversely, when individuals express an overall positive attitude toward vaccination, an ideologically biased writing style tends to lead them to perceive the information as more tentative than if it were presented by journalistic standards. The absence of an association between confidence and writing style and presentation layout suggests that varying how information is presented exerts relatively little influence on an individual’s confidence in COVID-19 vaccination. However, confidence remains closely linked to perceived tentativeness; individuals with high confidence levels in COVID-19 vaccines are less likely to perceive information as tentative.

The findings offer valuable insight into the potential significance of preexisting attitudes in processing and interpreting information on polarizing issues such as COVID-19 vaccination during a global health crisis [43]. This study suggests that deeply held beliefs significantly impact the effectiveness of informational efforts and that these beliefs are further reinforced when new information aligns with existing views. Preexisting attitudes toward vaccination appear to play a crucial role in shaping how individuals process and respond to vaccine-related information. It is thus essential not only to aim at countering misinformation but also to share clear and comprehensible information, ensuring it does not unnecessarily overload the target group’s cognitive processing while striving to understand better the underlying attitudes within the population to reach different audiences more effectively.

### Comparison With Previous Work

Misinformation about vaccines on web platforms raises public health concerns, including the escalation of vaccine hesitancy and the erosion of confidence [45,46]. The results of this study provide new insights into the role of hyperpartisan reinformation in shaping perceptions of COVID-19 vaccines. While a variety of approaches can be used to develop messages aimed at influencing behavior toward vaccination [47,48], a message founded on verifiable facts may not suffice to change the perspectives of individuals who hold antivaccine beliefs, as attitudes toward vaccination appear highly inflexible and strongly shape how people perceive and process information [49].

The pattern of results of the present study lends further empirical support to the effect of confirmation bias [50]. Grounded in the foundational work on cognitive dissonance theory [51,52], confirmatory information processing posits that individuals tend to resist or dismiss information conflicting with their beliefs [53,54]. The views of individuals on topics such as vaccination seem to be firmly fixed and difficult to change. Preexisting attitudes might have deep roots in personal beliefs about health and trust in health authorities or, more broadly, government and the public sphere. People with strong opinions often prefer evidence that supports their views rather than contradicts them [55,56]. The confirmation bias has been shown to impact the research and interpretation of information [56]. People with negative attitudes toward vaccination may view vaccine information skeptically, preferring information that aligns with their preferences. When dealing with issues that inherently generate disagreement among people, such as COVID-19 vaccination, polarization could lead to heightened levels of inflexibility, where individuals may not put in the cognitive effort to seek and process contradictory information. Preexisting attitudes may act as cognitive filters that influence how vaccine information is interpreted and trusted. In this way, cognitive dissonance theory and confirmation bias could be used to explain why individuals with strong antivaccine attitudes are resistant to new information.

Misinformation disseminated through reinformation media may influence individuals’ choices, aligning with their worldviews [57,58]. As highlighted by Baron [59], predisposed decisions are frequently intertwined with a certain level of overconfidence, potentially giving rise to the notion that others are more prone to being deceived by misinformation than ourselves [60]. In this case, maintaining consistency with beliefs about vaccination would prevent individuals from experiencing cognitive dissonance when presented with information about COVID-19 vaccines that contradicts their persona [40]. Such confirmation bias would be particularly strong because it would reinforce itself over time [61], making changing one’s attitude and beliefs toward vaccination even more challenging.

The elaboration likelihood model (ELM) [62] could also be relevant for explaining the limited impact of writing style on changing firmly established attitudes about divisive issues. According to the ELM, individuals process persuasive messages through 2 routes—the central route, which involves careful and thoughtful consideration of the arguments, and the peripheral route, which relies on superficial cues such as the credibility of the source or the attractiveness of the message. In the context of COVID-19 vaccination, where attitudes are deeply ingrained, the central route may be less engaged, limiting the effectiveness of the writing style and focusing instead on more substantial confirmatory arguments or evidence. As a result, the stylistic and layout aspects of the presentation may have only a minimal impact on altering rigid attitudes.

### Practical Recommendations

Public health communication strategies must address deep-seated beliefs and biases influencing perceptions, not just counteracting misinformation. One approach is for public health campaigns to segment their audience based on attitudes toward vaccination and tailoring messages accordingly. For individuals with positive attitudes toward vaccines, reinforcement messaging that confirms the safety and efficacy of vaccines could be effective in maintaining their confidence. For those with negative attitudes, messages that directly address common concerns and misconceptions, using empathetic and nonconfrontational language, may be more effective in reducing resistance and fostering openness to new information. Coupled with efforts to debunk false information, addressing misinformation individually could enhance the overall effectiveness of the campaign and improve public trust in accurate health information [63]. The present study highlights that changes in writing style and layout have a limited impact on altering attitudes, suggesting that merely varying the presentation of information is insufficient for effectively conveying public health messages and influencing public attitudes. Communication efforts could focus on identifying and partnering with trusted figures within communities, such as health care providers, local leaders, or influential social media personalities, who can deliver vaccine information in a credible and relatable manner [64]. Given the resilience of vaccine attitudes, it is also recommended to implement prebunking (preemptive refutation) strategies that inoculate individuals against misinformation before encountering it [65-67]. Public health campaigns can provide audiences with tools and frameworks to critically evaluate the credibility of information sources, helping them to recognize and reject misinformation. Educational content highlighting common tactics used in misinformation (such as emotional manipulation or false equivalence) can empower individuals to make more informed decisions [65-69]. Advocating for initiatives that promote critical thinking, that is, approaching real-world problems by considering the inherent properties of complex dynamic systems, could assist individuals in understanding the interconnections between factors that influence the emergence of an unforeseen situation requiring exceptional intervention, such as the global COVID-19 vaccination campaign to counteract the pandemic. As noted by Jackson [70], adopting a “critical systems thinking” approach to real-world issues provides an exciting avenue to comprehend complex and uncertain situations better and, concurrently, suggests which methodologies policy makers should use to craft effective educational policies that address misinformation before it significantly impacts people.

### Limitations

One limitation of the study derives from the sample composition. Our data collection was conducted among a nonprobability sample. Although the sample is representative of a range of sociodemographic characteristics of the Canadian population, the data set reported in the present paper does not include French-speaking Canadians, whether in Quebec or from Francophone minorities in other provinces. Logistical and sampling constraints combined with minimizing the potential for translation biases have led to the exclusion of data from the French-speaking Canadian population. However, further research is required to reflect the diversity of linguistic and cultural backgrounds better. It should also be noted that there was an overrepresentation of university students in the sample, which could be attributed to factors, such as accessibility or voluntary participation of candidates, on the Qualtrics panel system. Using a web-based panel excludes some population groups (eg, people without access to the internet). However, since our study was focused on misinformation on the web, this was not deemed a limitation. Finally, as with any survey, desirability bias cannot be excluded, but its impact should be minimal since the data collection was anonymous.

### Conclusions

Using a web-based randomized controlled trial experimental survey, we observed an association between the general attitude toward vaccination and the perception of COVID-19 vaccine information among English-speaking Canadians aged more than 18 years. Preexisting attitudes appear to play a significant role in COVID-19 vaccine hesitancy. When exposed to a source of reinformation sharing polarizing news about COVID-19 vaccination, provaccine individuals do not seem to be influenced beyond measure. The same rationale applies to vaccine skeptics; when assessing the tentativeness of a message, information about COVID-19 vaccines presented in a journalistic style fails to influence antivaccine individuals. The results also suggest that the perceived tentativeness of COVID-19 vaccine information is associated with confidence in COVID-19 vaccination.

Our findings are also significant for the broader context of public information dissemination, as they underscore how writing style and preexisting attitudes shape perceptions of credibility. Given the current prevalence of misinformation and biased reporting, this understanding is crucial for media outlets and is essential for enhancing effective communication with the public. Improving access to trustworthy and validated sources, identifying the nature of information outlets on the web, and promoting media literacy are critical factors in counteracting disinformation. Public health strategies that integrate tailored messaging, leverage trusted messengers, and implement prebunking techniques to address vaccine hesitancy are likely to be more effective. Such measures can help individuals develop the ability to identify instances when they encounter emotionally charged content or information that exhibits biases intended to endorse particular perspectives. Considering the complexity of vaccine hesitancy, a comprehensive multisectoral approach may be necessary, incorporating educational public policies and trust-building strategies that address the more considerable systemic barriers to vaccination. While the information is crucial, it alone is insufficient, and this may partially contribute to eroding the trust in the system for some individuals.

## Appendix

Multimedia Appendix 1 Questionnaires.

Multimedia Appendix 2 Artifact - journalistic writing style and journalistic layout with colored graphs.

Multimedia Appendix 3 Artifact - journalistic writing style and no journalistic layout.

Multimedia Appendix 4 Artifact - ideologically-biased writing style and no journalistic layout.

Multimedia Appendix 5 Artifact - ideologically-biased writing style and journalistic layout with colored graphs.

Multimedia Appendix 6 CONSORT e-HEALTH checklist (V 1.6.1).

Multimedia Appendix 7 Consent form.

## Abbreviations

CONSORT: Consolidated Standards of Reporting Trials
ELM: elaboration likelihood model
IdWJL: ideologically biased writing style and journalistic layout with colored graphs
IdWNL: ideologically biased writing style and no journalistic layout
IRRID: International Registered Report Identifier
JWJL: journalistic writing style and journalistic layout with colored graphs
JWNL: journalistic writing style and no journalistic layout

## Data Availability

The data sets generated during and/or analyzed during this study are available from the corresponding author on reasonable request.
