# Supplementary material for: The Resilience of Attitude Toward Vaccination: Web-Based Randomized Controlled Trial on the Processing of Misinformation
Source: JMIR Form Res. 2024 Dec 4;8:e52871. doi: 10.2196/52871 (PMC11656117; doi:10.2196/52871)
Supplement: Multimedia Appendix 1 [file formative_v8i1e52871_app1.pdf]

Start of Block: 1-Block_inclusion criteria

Q1_1 What is the highest level of school you have completed or the highest degree you have received?

- Less than high school degree (1)
- High school graduate (high school diploma or equivalent) (2)
- Some college but no degree (3)
- Associate degree in college (2-year) (4)
- Bachelor's degree in college/university (5)
- Master's degree (6)
- Doctoral degree (7)
- Professional degree (JD, MD) (8)

Page Break

Q1_2 Which age group do you fall into?

- ○ Less than 18 years old (10)
- ○ Between 18 and 24 years old (2)
- ○ Between 25 and 34 years old (3)
- ○ Between 35 and 44 years old (4)
- ○ Between 45 and 54 years old (5)
- ○ Between 55 and 64 years old (6)
- ○ Between 65 and 74 years old (7)
- ○ Between 75 and 84 years old (8)
- ○ 85+ years old (9)

Page Break

Q1_3 In which country do you currently reside?

▼ Afghanistan (1) ... Zimbabwe (1357)

Q1_4 Are you color-blind? In other words, do you have difficulty seeing colors?

- ○ Yes (1)
- ○ No (2)

Q1_5 What is your main reading language?

- ○ English (1)
- ○ French or other (5)

End of Block: 1-Block_inclusion criteria

Start of Block: Instructions

Q1_3 **Instructions** On the following screen, you will find a one-page document for you to read carefully. It is very important to take the time to read the entire document. Once you have finished reading the document, click on "next" to access the survey questions. You will not be able to go back and access the document again. It is therefore very important that you do not click on the "next" button until you have finished reading the document. Also, you will not be able to stop and save your answers in order to complete the survey later. You must complete all tasks in one step from start to finish. Click on “Next” to access the document.

End of Block: Instructions

Start of Block: 2-Block_arm1_standard

Q120

Q111 Timing
First Click (1)
Last Click (2)
Page Submit (3)
Click Count (4)

End of Block: 2-Block_arm1_standard

Start of Block: 3- Block_arm2

Q123

Q112 Timing
First Click (1)
Last Click (2)
Page Submit (3)
Click Count (4)

End of Block: 3- Block_arm2

Start of Block: 4- Block_arm3

Q128

Q113 Timing
First Click (1)
Last Click (2)
Page Submit (3)
Click Count (4)

End of Block: 4- Block_arm3

Start of Block: 5- Block_arm4

Q130

Q114 Timing
First Click (1)
Last Click (2)
Page Submit (3)
Click Count (4)

End of Block: 5- Block_arm4

Start of Block: 6- Adapted NASA-TLX

Q6_1 How hurried or rushed was the pace of the task (i.e., reading the document)?

	Very low	Very high
	0 1 2 3 4 5 6 7 8 9 10 11 12 13 14 15 16 17 18 19 20	
1 ()		

Q6_2 Which of the following statements best describes the topic of the document you have just read?

- ○ Lockdown of unvaccinated people (1)
- ○ Measles vaccine side effects (2)
- ○ COVID-19 and school closures (3)
- ○ COVID-19 vaccine side effects (4)
- ○ COVID-19 and loss of smell (5)

End of Block: 6- Adapted NASA-TLX

Start of Block: 7- Outcome measures

Page Break

Page Break

Q7_3 The information in the document is definitive.

- Strongly disagree (1)
- Disagree (2)
- Neither agree nor disagree (4)
- Agree (6)
- Strongly agree (7)

Q7_4 Based on this document, our understanding of COVID-19 vaccine side effects is complete.

- Strongly disagree (1)
- Disagree (2)
- Neither agree nor disagree (4)
- Agree (6)
- Strongly agree (7)

Q7_5 The document is conclusive.

- Strongly disagree (1)
- Disagree (2)
- Neither agree nor disagree (4)
- Agree (6)
- Strongly agree (7)

Q7_6 The information reported in the document is reliable.

- Strongly disagree (1)
- Disagree (2)
- Neither agree nor disagree (4)
- Agree (6)
- Strongly agree (7)

Q7_7 The document provides a strong basis for deciding whether or not to take the COVID-19 vaccine in the future.

- Strongly disagree (1)
- Disagree (2)
- Neither agree nor disagree (4)
- Agree (6)
- Strongly agree (7)

Q7_6 The information reported in the document should not be considered preliminary.

- Strongly disagree (1)
- Disagree (2)
- Neither agree nor disagree (4)
- Agree (6)
- Strongly agree (7)

Q7_7 The information reported in the document is credible.

- Strongly disagree (1)
- Disagree (2)
- Neither agree nor disagree (4)
- Agree (6)
- Strongly agree (7)

Q116 Timing
First Click (1)
Last Click (2)
Page Submit (3)
Click Count (4)

Page Break

Q7_2_1 COVID-19 vaccine is promising.

- Strongly disagree (1)
- Disagree (2)
- Neither agree nor disagree (4)
- Agree (6)
- Strongly agree (7)

Q7_2_2 COVID-19 vaccine is safe.

- Strongly disagree (1)
- Disagree (2)
- Neither agree nor disagree (4)
- Agree (6)
- Strongly agree (7)

Q7_2_3 COVID-19 vaccine is certainly helpful.

- Strongly disagree (1)
- Disagree (2)
- Neither agree nor disagree (4)
- Agree (6)
- Strongly agree (7)

Q7_2_4 The risks related to COVID-19 vaccine are lower than the benefits.

- ◯ Strongly disagree (1)
- ◯ Disagree (2)
- ◯ Neither agree nor disagree (4)
- ◯ Agree (6)
- ◯ Strongly agree (7)

Q7_2_5 If a loved one had a need for which COVID-19 vaccine is one of the solutions, I would like him or her to benefit from it.

- ◯ Strongly disagree (1)
- ◯ Disagree (2)
- ◯ Neither agree nor disagree (4)
- ◯ Agree (6)
- ◯ Strongly agree (7)

Q7_2_6 If I had a need for which COVID-19 vaccine is one of the solutions, I would like to benefit from it.

- ◯ Strongly disagree (1)
- ◯ Disagree (2)
- ◯ Neither agree nor disagree (4)
- ◯ Agree (6)
- ◯ Strongly agree (7)

Q7_2_7 COVID-19 vaccine roll-out is not concerning.

- ◯ Strongly disagree (1)
- ◯ Disagree (2)
- ◯ Neither agree nor disagree (4)
- ◯ Agree (6)
- ◯ Strongly agree (7)

Q7_2_8 Currently available vaccines are the most effective way to combat the COVID-19 epidemic.

- ◯ Strongly disagree (1)
- ◯ Disagree (2)
- ◯ Neither agree nor disagree (4)
- ◯ Agree (6)
- ◯ Strongly agree (7)

Q117 Timing
First Click (1)
Last Click (2)
Page Submit (3)
Click Count (4)

Page Break

End of Block: 7- Outcome measures

Start of Block: 8- Individual attributes and previous knowledge

Q8_1 Here is a 10-point scale on which the views that people might hold are arranged from extremely pro-vaccine (1) to extremely anti-vaccine (10). Where would you place yourself on this scale?

	Extremely pro-vaccine					Extremely anti-vaccine				
	1	2	3	4	5	6	7	8	9	10
1 ()										

Page Break

Q8_2 Is COVID-19 vaccine in your field of professional expertise?

○ Yes (1)

○ No (2)

Page Break

Q8_3 What sex were you assigned at birth?

- Male (1)
- Female (2)
- Intersex (3)
- Prefer not to disclose (4)

Q8_4 Please indicate which of the following occupation groups best describes your current occupation. If you are retired, please indicate the group that best describes your occupation prior to retirement. If you are unemployed, please indicate the group that best corresponds to the job you had before you left or lost your job.

- ◯ Architecture and Engineering (1)
- ◯ Arts and Design (2)
- ◯ Building and Grounds Cleaning (3)
- ◯ Business and Financial (4)
- ◯ Community and Social Service (5)
- ◯ Computer and Information Technology (6)
- ◯ Construction and Extraction (7)
- ◯ Education, Training, and Library (8)
- ◯ Entertainment and Sports (9)
- ◯ Farming, Fishing, and Forestry (11)
- ◯ Food Preparation and Serving (12)
- ◯ Healthcare (13)
- ◯ Installation, Maintenance, and Repair (14)
- ◯ Legal (15)
- ◯ Life, Physical, and Social Science (16)
- ◯ Management (17)
- ◯ Math (18)
- ◯ Media and Communication (19)
- ◯ Military (20)

- ○ Office and Administrative Support (21)
- ○ Personal Care and Service (22)
- ○ Production (23)
- ○ Protective Service (24)
- ○ Sales (25)
- ○ Transportation and Material Moving (26)

Page Break
