# Supplementary material for: The Resilience of Attitude Toward Vaccination: Web-Based Randomized Controlled Trial on the Processing of Misinformation
Source: JMIR Form Res. 2024 Dec 4;8:e52871. doi: 10.2196/52871 (PMC11656117; doi:10.2196/52871)
Supplement: Multimedia Appendix 5 [file formative_v8i1e52871_app5.pdf]

Alternative News of Today

COVID-19 vaccines: side effects, benefits and risks

News · Posted: Feb 01, 2022 8:00 AM ET | Last Updated : 15 minutes ago

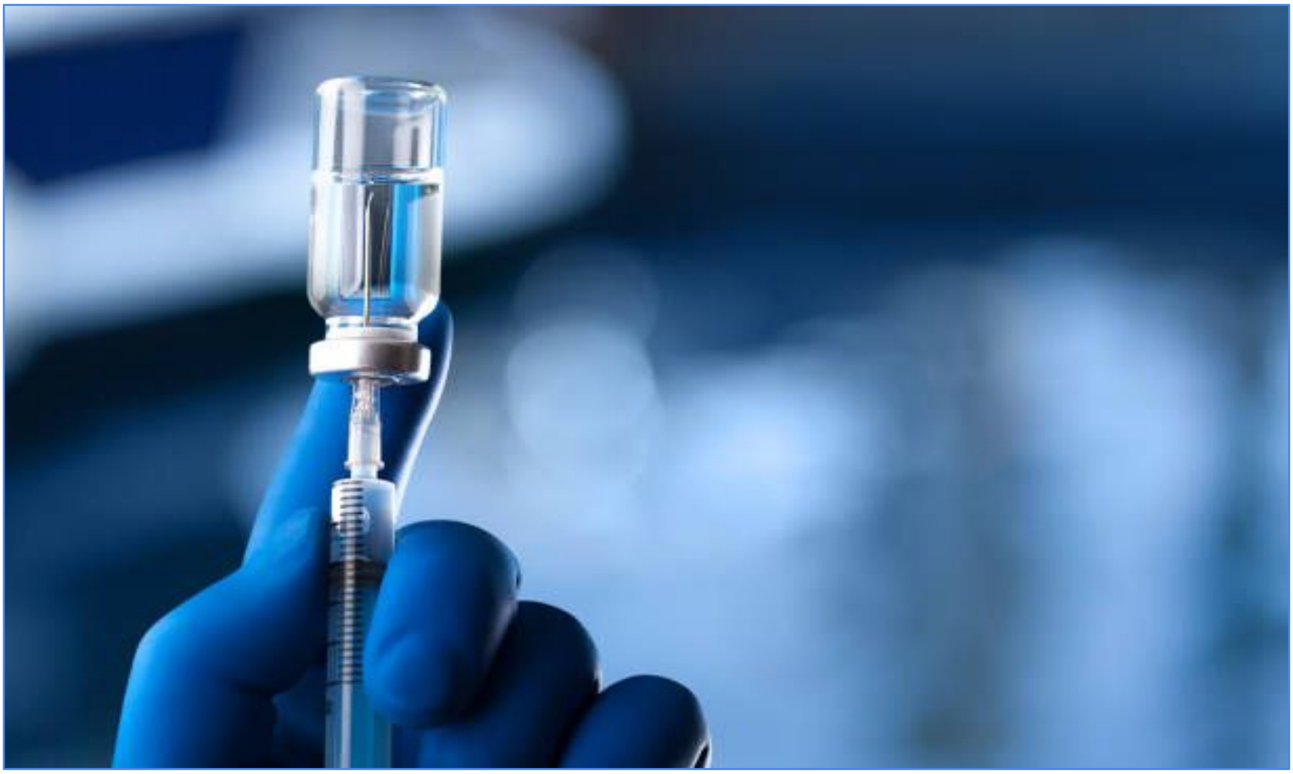

Images via IStock; Credits Alernon77

Vaccination against The Wuhan Flu is still recommended by scientists despite risks of myocarditis and other secondary effects! Yet, according to one of the latest study and the largest of its kind to be conducted on potential side effects of receiving mRNA vaccines, the risk of developing myocarditis, usually a very rare inflammation of the heart muscle, may be greater from contracting Covid-19 than from receiving the vaccine…

In December, some researchers from the University of Oxford compared the risks of cardiovascular events, such as myocarditis, pericarditis, and cardiac arrhythmia, between different vaccines as well as COVID-19 infection. Their study, published in *Nature Medicine*, looked at data from 38 million people aged 16 or older vaccinated for COVID-19 in England between December 1, 2020 and August 24, 2021. **Their** conclusion is that the number of cases of myocarditis was at least four times greater after Covid-19.

In the opinion of Julia Hippisley-Cox, a professor of clinical epidemiology and general practice at Oxford and study lead of this research, "*COVID-19 vaccines are highly effective at reducing risks of severe outcomes from COVID-19 infection. And what [we've] found here continues this finding*".

“*Whilst there are some increased risks of rare heart related complications associated with vaccines these are much lower than the risk associated with getting COVID-19*,” she keeps explaining!

Indeed, a link between heart inflammation and vaccination was not observed during **clinical trials** of the most common COVID-19 vaccines, but shortly after vaccines began rolling out across the globe concerns are being raised. Earlier reports from the United States and Israel found **some adolescents and young adults, especially men and boys, developed myocarditis after receiving mRNA vaccines**!!! More than one study has found risks associated with the second dose of the Moderna vaccine, in particular, such as a very much higher risk of arrhythmia.

“*It is important that we know about and identify the risks of these rare conditions from vaccines as well, to ensure that clinicians know what to look for, aid earlier diagnosis, and inform clinical decision making and resource management*,” said Hippisley-Cox herself.

Symptoms of myocarditis include chest pain, breathlessness and heart palpitations. They can be mild or severe and can lead to permanent heart damage and even death. However, they say extreme forms are rare and most cases can be treated, according to them, with over-the-counter anti-inflammatory drugs, such as ibuprofen!

However, these researchers admit themselves there were limitations in this study and young people aged 16 to 40 were underrepresented in the sample! This can be very discouraging for parents who wrestle with the decision to get their children vaccinated or not… About one in 300,000 children who test positive for Covid-19 die, according to data from the UK government. While the risk of being hospitalized is also low, children with serious underlying conditions remain vulnerable and children may also pass the virus on to other vulnerable adults. Governments say they were eager to begin vaccinating children as soon as the vaccines were approved for this reason… Thus, the European Union's medicines regulator approved the use of the Pfizer-BioNTech vaccine for five to 11-year-old’s at the end of November, followed shortly by the US Food and Drug Administration and Health Canada!

Nicholas Mills, a consultant cardiologist and professor of cardiology at the Centre for Cardiovascular Science at the University of Edinburgh tried to reassure citizens and lawmakers concerned by reports about possible vaccine side effects in young people.

“**We're** not seeing any data here that would change the recommendation on vaccinating children,” he told the *Daily Mail*…
