# Supplementary material for: The Resilience of Attitude Toward Vaccination: Web-Based Randomized Controlled Trial on the Processing of Misinformation
Source: JMIR Form Res. 2024 Dec 4;8:e52871. doi: 10.2196/52871 (PMC11656117; doi:10.2196/52871)
Supplement: Multimedia Appendix 7 [file formative_v8i1e52871_app7.pdf]

FACT SHEET FOR IMPLIED AND CONFIDENTIAL CONSENT

TITLE OF RESEARCH: Analysis of sensemaking through the presentation of written style-induced bias information in a news article on COVID-19 vaccination

MAIN RESEARCHERS: Sébastien Tremblay (School of Psychology) and Mathieu Ouimet (Department of Political Science) from Université Laval.

INFORMATION ABOUT THE PROJECT:
Besides its significant social impacts, particularly on public health, the COVID-19 pandemic has generated a significant infodemic on both digital platforms and traditional media. Several scientific studies demonstrate that the human capacity to adequately manage informational complexity is limited (e.g., Cronin, Gonzalez, & Sterman, 2009; Koechlin & Hyafil, 2007). In the current pandemic context, information is overabundant, creating a complex environment in which consumers - as well as producers and disseminators of information - are vulnerable to decision bias and disinformation.

YOUR PARTICIPATION:
Your participation in this research will consist of filling this questionnaire that includes questions about a news article on COVID-19' secondary effects. Although the answers to each question are important for our research, you remain free not to answer any of those questions or to terminate your participation at any time without having to provide any justification. At the end of the questionnaire, you will be asked if you wish to submit your questionnaire and participate in our study. Only in this option will your questionnaire be considered filled and completed. If you consent, please note that it will not be possible to remove your data from the study. Your data will be retained according to the methods described below, which will be applied to all participants.

COMPENSATION:
If you are reading this document, you have already agreed to receive invitations to participate in surveys administered by Qualtrics pending compensation. Qualtrics does not give direct payment to the participants, but give points that equal monetary value such as credit card rewards program where you collect points and are able to put those toward vacation, travel, gifts, etc. You will be compensated the amount you agreed upon before you entered into the survey. If you complete the study, but do not give a quality response (e.g., gibberish for open-ended questions, select the first answer choice for each question, or speed through the survey), you are not compensated. To receive the compensation, you need to complete the entire study. Partial completion does not receive compensation.

CONFIDENTIALITY
Researchers are required to ensure participants' confidentiality. In this regard, all gathered data are anonymous. Here are the measures that will be applied within the framework of our research:

- The research team will not possess any information on your identity;
- The research material will be store on a computer located at the Université Laval in a secured location and will destroyed by June 2024;
- Data in digital format will be stored in encrypted, password-protected files whose access

will be restricted to the researcher only;

- After the research, data will be stored for future use in a definitively irreversible coded and aggregated form (i.e. grouped by category with data from other participants) in an anonymous database.

During dissemination of results:

- The results will be presented in aggregate form so that the individual results of the participants will never be divulgated;
- The research results will be published in the form of scientific publications and administrative reports in which no participant can be identified.

ACKNOWLEDGMENTS:
Your invaluable collaboration makes it possible for us to carry out this study. That is why we would like to thank you for the time and attention you dedicate to your participation in this research.

PROOF OF CONSENT:
Returning the filled questionnaire will be considered as the implicit expression of your consent to participate in the project.

ADDITIONAL INFORMATION:
If you have any questions about the research, the implications of your participation, if you wish to receive a short summary of the research results, please contact Sébastien Tremblay, Sebastien.Tremblay@psy.ulaval.ca

COMPLAINTS OR CRITICAL COMMENTS:
Any complaint or critical comment pertaining to your participation in this research project may be addressed confidentially to the Office of the Ombudsman at Université Laval:

Pavillon Alphonse-Desjardins, office 3320
2325 Rue de l'Université
Université Laval
Quebec City (Quebec) G1V 0A6
Information – Secretariat: (418) 656-3081
Toll-free line: 1-866-323-2271
Email: info@ombudsman.ulaval.ca
